# A Decade of Focus on and Improvement in Access to Care in the Veterans Health Administration

**DOI:** 10.1007/s11606-023-08208-1

**Published:** 2023-06-20

**Authors:** Peter J. Kaboli, Stephanie L. Shimada

**Affiliations:** 1 Veterans Rural Health Resource Center-Iowa City, VA Office of Rural Health, and Center for Access and Delivery Research and Evaluation (CADRE) at the Iowa City VA Healthcare System, Iowa City, IA USA; 2 Department of Internal Medicine, University of Iowa Carver College of Medicine, Iowa City, IA USA; 3 Center for Healthcare Organization and Implementation Research, VA Bedford Healthcare System, Bedford, MA USA; 4 Department of Health Law, Policy and Management, Boston University School of Public Health, Boston, MA USA; 5 Division of Health Informatics and Implementation Science, Department of Population and Quantitative Health Sciences, University of Massachusetts Chan Medical School, Worcester, MA USA

The Veterans Administration (VA) has the largest integrated healthcare system in America serving over 9 million Veterans across almost 1300 healthcare facilities.^1^ Providing Veterans access to timely and high-quality care has always been a key strategic priority driven by VA’s mission.^2^ In 2011, *JGIM* published a special supplement *Improving Access to VA Care*^3^ to categorize the then current state of access research, a reconceptualization of access (see Fortney model in Fig. 1)^4^, and report findings from a VA State of the Art Conference on Improving Access to Care. In the last decade, Congress has passed significant legislation to shape and improve how Veterans access healthcare. Most notably, the Veterans Access, Choice and Accountability Act of 2014, the VA Maintaining Internal Systems and Strengthening Integrated Outside Networks Act (MISSION) Act of 2018, and the Cleland-Dole Act of 2022 have provided Veterans with choices in their communities and shorter wait-times. As evidenced by the 140 submissions received from the Call for Papers for this access supplement, VA researchers continue to be hard at work supporting VA’s mission. In this new supplement, we highlight recent advancements in access-related research and how VA has evolved in its ongoing journey to be a high-reliability organization using a learning healthcare system framework to improve access to care.

**Figure 1:**
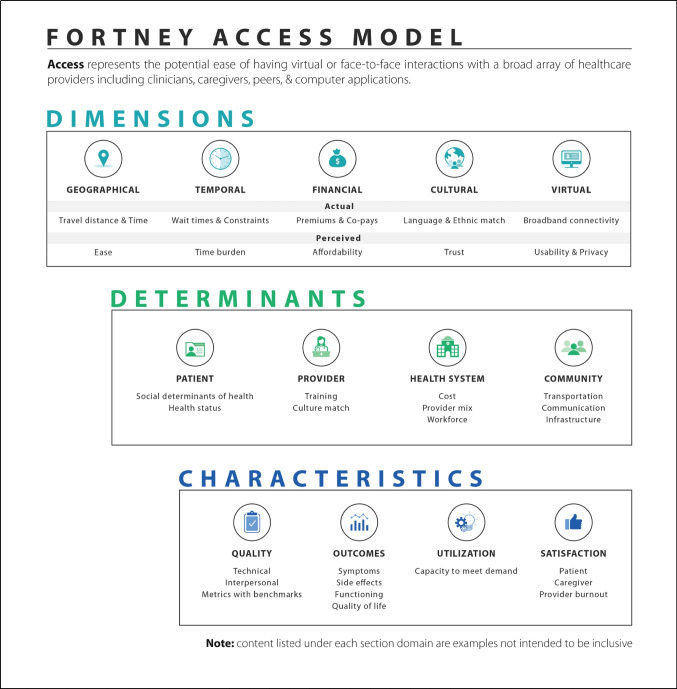
Fortney access model.

The Veterans Access Research Consortium, a VA Health Services Research and Development Consortium of Research (CORE), sought input from a network of access researchers, operational leaders, and Veterans to shape research priorities for healthcare access.^5^ This supplement built on that work by soliciting submissions aligned with three domains identified as being the highest priority for VA access research:

1. **Access Measurement**: How should actual and perceived access be defined and measured so it is understandable, uses the best possible data (e.g., surveys, electronic), and has meaningful implications for Veteran outcomes, both in VA and the community?
2. **Equity and Subpopulations**: How can we ensure equitable and effective access to services for Veterans who are underrepresented or experience disparities in VA (e.g., racial/ethnic minority individuals, people who identify as LGBTQ + , women, Native Americans)?
3. **Effective Interventions to Improve Access**: What are the most effective and scalable interventions that improve access, considering different modalities (e.g., in-person, virtual), settings (e.g., VA, community), and targets (e.g., patients, providers, system)?

## ACCESS MEASUREMENT

A key principle of learning healthcare systems is that continual assessment is required to improve processes and care; thus, measurement has been and will continue to be a persistent need in access research. Two papers address the geographical access dimension through excess travel burden for sleep apnea testing (Hahn et al.) and identifying women Veterans who have excess drive times for obstetric referrals (Vinekar et al.). Patzel et al. report on the challenges of non-VA primary care providers who care for Veterans and address three determinants of access: provider, health system, and community perspectives. They found that effective communication can reduce confusion, clarify responsibilities, facilitate medical record sharing, and help navigate care for Veterans across multiple systems. Lastly, Smith et al. provide an analytic approach to psychiatry staffing models to address the health system workforce, a key determinant of access. Together, these and other studies in this issue have advanced our tools to measure access and identify actionable targets to guide improvement.

## EQUITY AND SUBPOPULATIONS

Special attention is required to ensure VA provides equitable and effective access to services without imposing new or unexpected barriers for Veteran subpopulations. Several papers in this issue address important equity challenges among special populations: the digital divide (O’Shea et al., Goldstein et al.), homeless Veterans (McCoy et al.), unmet social needs (Gurewich et al.), LGBTQ + Veterans, (Wilson et al.), and women Veterans (Mahorter et al., Goldstein et al.). Some help us identify the challenges members of these subgroups face in healthcare, while others offer innovative solutions to improving health inequities. Telemedicine is an important tool to improve access and overcome geographic and temporal barriers to care. However, it is not without risk of exacerbating the digital divide, requiring those with inadequate access to use in-person visits or potentially forego care when virtual visits may have substituted. McCoy et al. describe lessons learned from a sanctioned encampment to provide housing and healthcare to Veterans experiencing homelessness during the pandemic at the Greater Los Angeles VA, an innovative and proactive approach to one of society’s most vexing challenges. Mahorter et al.’s report on access to long-acting reversible contraception demonstrates the importance of provider training as a determinant of access to reproductive health services, and the importance of identifying and addressing variations in access that can exacerbate health outcomes in an important Veteran subpopulation.

## INTERVENTIONS

Ultimately, interventions are needed to show how we improve access. In a randomized controlled trial (RCT) of a “PTSD Coach” mobile app with mental health clinician support, Possemato et al. demonstrated expanded geographic access, improved temporal access, and addressed a key determinant — provider capacity to deliver evidence-based mental health. This RCT improved access and quality, outcomes, and satisfaction for Veterans with PTSD. Teo et al. used a pragmatic trial to test behavioral economics–informed reminders to reduce missed appointments. Their study demonstrated the difficulty of eliminating no-shows and serves as a valuable reminder of the importance of publishing null findings to inform future interventions. Cusick et al. reported on a regional initiative to coordinate care for Veterans across two healthcare systems that resulted in more continuity within VA for patients and improved efficiency. Arredondo et al.’s narrative review of programs and incentives to overcome rural physician shortages addresses provider and health system determinants of access. What is still lacking is understanding the efficacy of such incentives to fill both short-term staffing challenges and long-term workforce needs. Lastly, Dodge et al. highlight the paramount importance of partnerships between researchers and operational offices to ensure that effective access-related interventions are implemented into everyday practice.

Impactful medical research advances our scientific knowledge while also shaping clinical care and policy. The research findings in this supplement help move the field forward in the following ways:

1. **Continue access metric development, validation, and reporting**. Learning healthcare systems leverage data and measurement to ensure they meet patient needs and optimize resource use. Researchers must continue to work with key operational partners to determine access metrics beyond wait times that can be used across populations, conditions, and healthcare settings to identify actionable targets for interventions and monitor equity and progress.
2. **Build on successes and learn from failures**. Virtual care has successfully revolutionized access to healthcare both within VA and beyond.^6^ This supplement includes interventions that demonstrate VA’s innovative spirit, such as care coordination initiatives for Veterans experiencing homelessness. Exploring variations in quality and access may highlight failures, but it can also identify high value care to serve as best practices. Likewise, research with null findings can help redirect research efforts and avoid expanding unproven interventions.
3. **Virtual care needs ongoing adoption, but with vigilance to ensure equitable access to high quality care for all, including vulnerable subpopulations**. Expanding virtual care options through video telehealth, specialty consultation, e-consults, and mobile apps has great potential. Research consistently shows virtual health can provide similar outcomes with improved access for a variety of medical conditions.^6^ However, we must carefully monitor quality as we continue to ascertain when virtual care is superior, equivalent, or inferior to in-person care based on a patient’s needs, preferences, medical conditions, or membership in vulnerable subgroups. Real concerns remain of the digital divide widening disparities for some subgroups if we do not monitor access measures.

## CONCLUSIONS

While preparing this supplement, we reflected on how much access-related measurement, interventions, and equity have improved in VA since the initial *JGIM* “Improving Access to VA Care” supplement in 2011. Wait time and quality metrics are systematically made available to patients online^7^ and a more expansive collection of access measures have been made available by and for VA researchers. However, longitudinal tracking and reporting need to be leveraged to clarify where VA is already achieving optimal and timely access, and where we should be targeting improvement. These measurement advances facilitate studies of interventions, many of which have shown robust local, regional, and national improvements in access. Further translational efforts are needed to determine the cost- and comparative effectiveness of promising access interventions and implement them into practice. Since 2011, Veterans are increasingly included as partners in research to ensure that they have a voice in shaping interventions that impact their access. VA has significantly increased its services targeting subpopulations of Veterans (e.g., LGBTQ + , women, and homeless Veterans), and this is reflected in VA’s research portfolio. The focus on equity and subpopulations has identified areas of concern such as the digital divide to potentially prevent worsening inequity. The COVID-19 pandemic exponentially increased uptake of telehealth modalities, increasing overall access while demanding appropriate attention to avoiding growing inequities in access and outcomes.^8^

For decades, the VA has been investing in strengthening its capacities as a learning healthcare system.^9^ Partnerships between clinicians, leadership, researchers, Veterans, and national program offices support meaningful improvements in access to quality care for all Veterans. By continuing on this path with increasing transparency in how we measure, report, and improve access, VA has the potential to serve as the standard by which other healthcare systems compare themselves. The work in this supplement and the many other access-focused VA studies being conducted are a testament to VA’s ongoing dedication to a continuously learning health system.
